# HIV serologic reactivity varies with time of ART initiation in persons on long-term ART

**DOI:** 10.1128/jcm.01273-25

**Published:** 2025-12-17

**Authors:** Vivian I. Avelino-Silva, Mars Stone, Clara Di Germanio, Marion C. Lanteri, Sonia Bakkour, Eduard Grebe, Brian Custer, Xutao Deng, Satish Pillai, Renata Buccheri, Steven H. Kleinman, Sandhya Vasan, Morgane Rolland, Nittaya Phanuphak, Carlo Sacdalan, Siriwat Akapirat, Mark de Souza, Esper G. Kallas, Sheila de Oliveira Garcia Mateos, Ester C. Sabino, Michael P. Busch, Philip J. Norris, for the NHLBI Recipient Epidemiology and Donor Evaluation Study-IV-Pediatric (REDS-IV-P)

**Affiliations:** 1 Vitalant Research Institute 166672, San Francisco, California, USA; 2 Department of Epidemiology and Biostatistics, University of California San Francisco 166607, San Francisco, California, USA; 3 Faculdade de Medicina da Universidade de Sao Paulo 37884 , São Paulo, Brazil; 4 Department of Laboratory Medicine, University of California San Francisco 8785, San Francisco, California, USA; 5 Creative Testing Solutions, Tempe, Arizona, USA; 6 University of British Columbia 8166, Victoria, British Columbia, Canada; 7 U.S. Military HIV Research Program, Walter Reed Army Institute of Research 8394, Silver Spring, Maryland, USA; 8 Henry M. Jackson Foundation for the Advancement of Military Medicine, Inc., Bethesda, Maryland, USA; 9 Institute of HIV Research and Innovation (IHRI) 606508, Bangkok, Thailand; 10 SEARCH Research Foundation, Bangkok, Thailand; 11 Research Affairs, Faculty of Medicine, Chulalongkorn University 65103, Bangkok, Thailand; 12 Armed Forces Research Institute of Medical Sciences (AFRIMS) 544411, Bangkok, Thailand; 13 Instituto Butantan 196591, São Paulo, Brazil; 14 Universidade Municipal de Sao Caetano do Sul 119501, São Caetano do Sul, Brazil; 15 Department of Medicine, University of California San Francisco 166668, San Francisco, California, USA; St Jude Children's Research Hospital, Memphis, Tennessee, USA

**Keywords:** HIV testing, serologic tests, sensitivity and specificity, highly active antiretroviral therapy, blood donation

## Abstract

HIV serologic responses measured by clinical screening assays may be blunted when antiretroviral treatment (ART) suppresses HIV replication from the initial infection stages, with further decay following long-term treatment. Sensitivity may vary according to the timing of ART initiation after HIV acquisition, antigens (Ags) utilized to detect anti-HIV antibodies (Abs), and HIV subtype. We obtained samples from long-term ART-suppressed persons with HIV (PWH) who started ART at acute/early or during chronic infection, from blood donors with undetectable HIV RNA and nonreactive serology, and from blood donors with positive RNA and reactive serology (presumably untreated infections) spanning multiple HIV subtypes to compare patterns of serologic reactivity using two Ag/Ab screening assays (Alinity and VITROS). We found that earlier ART initiation was associated with progressively lower signal-to-cutoff ratios (S/CO) in both assays. Serologic reactivity in clinical samples from participants initiating ART at Fiebig 2 was somewhat lower for CRF01_AE (67%) than other HIV subtypes (91%) on the Alinity platform. In the late-ART initiation group, reactivity was high for both assays despite long-term treatment, with higher S/CO values in samples from blood donors with presumably untreated infection. We observed no strong evidence of S/CO waning >96 weeks after ART initiation in the early-ART initiation group. Our findings suggest potential limitations for HIV infection ascertainment in clinical samples from long-term treated PWH who started ART at early infection stages using currently approved serologic tests.

**IMPORTANCE:**

This manuscript increases our understanding of how the timing of initiation of HIV treatment affects our ability to detect the infection using commercial blood tests that measure HIV antigens or antibodies. Being able to detect HIV is important for clinical diagnostics and blood screening, and HIV is not as easily detected in persons who begin therapy shortly after infection using serological tests. In contrast to prior work, we show that current clinical HIV tests have stable reactivity over time in persons on long-term HIV treatment.

## INTRODUCTION

Serologic tests have been implemented for HIV screening and diagnosis since 1985, initially detecting IgG antibodies (Abs), subsequently incorporating IgM, and finally combining p24 antigen (Ag) detection. This allowed increasingly higher sensitivity to detect infections earlier, reducing the window/eclipse period (1, 2). Serologic tests are a key component of blood screening testing algorithms, particularly for detecting donations from persons with undisclosed HIV infection on antiretroviral therapy (ART) whose samples may be negative by nucleic acid testing (NAT) but reactive by serology testing (3).

Blunted serologic reactivity for HIV has been shown among HIV pre-exposure prophylaxis (PrEP) users with breakthrough infections (4–7) and among persons with HIV (PWH) treated since acute/early infection (4, 8, 9). Most early ART studies have focused on blood samples collected in the first weeks or months after treatment initiation, but a decline in HIV Abs may occur several years after ART initiation (10–12). While studies failed to show major subtype-specific limitations across multiple Ag/Ab combination assays (13–15), it is plausible that subtle differences in reactivity may become apparent in samples obtained from persons with blunted Ab responses.

In this study, we obtained samples from cohorts of long-term ART-suppressed PWH who started ART at acute/early or during chronic infection, from blood donors who tested negative by NAT and nonreactive by serology, and from blood donors who tested positive by NAT and reactive by serology during blood donation screening (presumably untreated infections) to compare the patterns of serologic reactivity as measured and analyze trends over time among PWH after long-term treatment.

## MATERIALS AND METHODS

### Study panels and samples

We obtained longitudinal samples from ART-suppressed PWH who started ART at acute/early stages of infection (early-ART initiation group) from RV254/SEARCH 010 (RV254) (16), a prospective cohort study conducted in Bangkok, Thailand. At the cohort entry, parallel NAT and serology screening allowed classification of each participant according to the Fiebig stage (17) at ART initiation (18). We obtained plasma samples collected at early time points (0, 12, and 24 weeks) for 55 participants as well as available later time points spanning 96–480 weeks after ART initiation. In addition, we tested all available samples from a larger sample of RV254 participants at week 336 (*n* = 300) and week 480 (*n* = 75) after ART initiation. For comparison, we also included the following cross-sectional samples:

– Plasma samples from blood donors identified as HIV NAT-negative and HIV serology nonreactive (RNA−/serology−) obtained from US Vitalant donations. Routine screening for Vitalant blood donations includes parallel HIV NAT testing in minipools of 16 using the Procleix Ultrio Elite assay (Grifols Diagnostic Solutions) and individual serology testing using the Alinity s HIV Ag/Ab Combo Reagent Kit assay (Abbott Laboratories). A panel of 50 donation samples was initially selected for study testing. De-identified results from routine donation screening tests done in 373,215 additional samples were obtained for the analysis of test sensitivity at different test cutoffs;
– Plasma samples from 50 PWH who started ART after fully established infections (late-ART initiation group), recruited at the University of Sao Paulo Medical School’s HIV outpatient clinic in Brazil;
– Serum samples from 50 blood donors identified as HIV-1 RNA-positive with viral load >1,000 copies/mL and HIV serology reactive (RNA+/serology+; presumably untreated infections), obtained from the Recipient Epidemiology and Donor Evaluation Study-IV-Pediatric (REDS-IV-P) Brazil program. Blood centers in the REDS-IV-P Brazil program routinely screen blood donations using a fourth-generation HIV-1/2 Ag/Ab combination test in parallel with HIV-1 NAT in minipools of six (19).

### Laboratory assays

All samples were tested with two commercially available Ag/Ab combination assays previously approved by the US Food and Drug Administration: the Alinity s HIV Ag/Ab Combo Reagent Kit (Abbott Laboratories; https://www.fda.gov/vaccines-blood-biologics/approved-blood-products/alinity-s-hiv-agab-combo-assay), currently approved for blood screening, and the VITROS Immunodiagnostic Products HIV Combo Reagent Pack (Ortho-Clinical Diagnostics; https://www.fda.gov/vaccines-blood-biologics/approved-blood-products/vitros-immunodiagnostic-products-hiv-combo-reagent-pack-vitros-immunodiagnostic-products-hiv-combo), currently approved for HIV diagnosis in clinical settings. Both Alinity and VITROS are used for simultaneous qualitative detection of HIV p24 Ag and Abs to HIV types 1 and 2 in serum or plasma, providing signal-to-cutoff ratios (S/CO) indicating the sample’s signal strength relative to the test cutoff. Although all tests were performed according to the manufacturers’ instructions, due to study design and logistical considerations, sample collection, processing, and storage prior to testing were not always performed according to Alinity or VITROS assay package insert requirements. For example, some of the RV254 samples were stored at ≤−20°C, beyond labeled claims.

Assessment of the HIV subtype had been performed as part of RV254 using the multiregion subtype-specific PCR (MSSP) assay as previously described (20) for 78% of the samples and sequencing for 22% of the samples. HIV-1 sequences were obtained either as a near full-length genome or as two half genomes overlapping by approximately 1.5 kb by single-genome sequencing, as previously described (21, 22).

### Statistical analysis

We compared S/CO values between groups using Wilcoxon-Mann-Whitney rank-sum tests. Correlations between S/CO values and time since ART initiation were investigated using the Spearman correlation test. For longitudinal assessment of S/CO values in RV254 participants, linear mixed-effects models with a random intercept for participants were used to assess overall trends in assay signal. Among participants in the late-ART initiation group, six had incomplete information on the date of ART initiation (only year available for four and only month/year available for two). In these cases, we imputed the date of ART initiation as the midpoint of the available date to estimate the number of weeks on ART at sample collection. For all analyses, we used Stata version 17 (StataCorp LP) with a 0.05 significance level. Figures 2, 3, and 5 were created using R (https://www.r-project.org/).

## RESULTS

**Fig 1:**
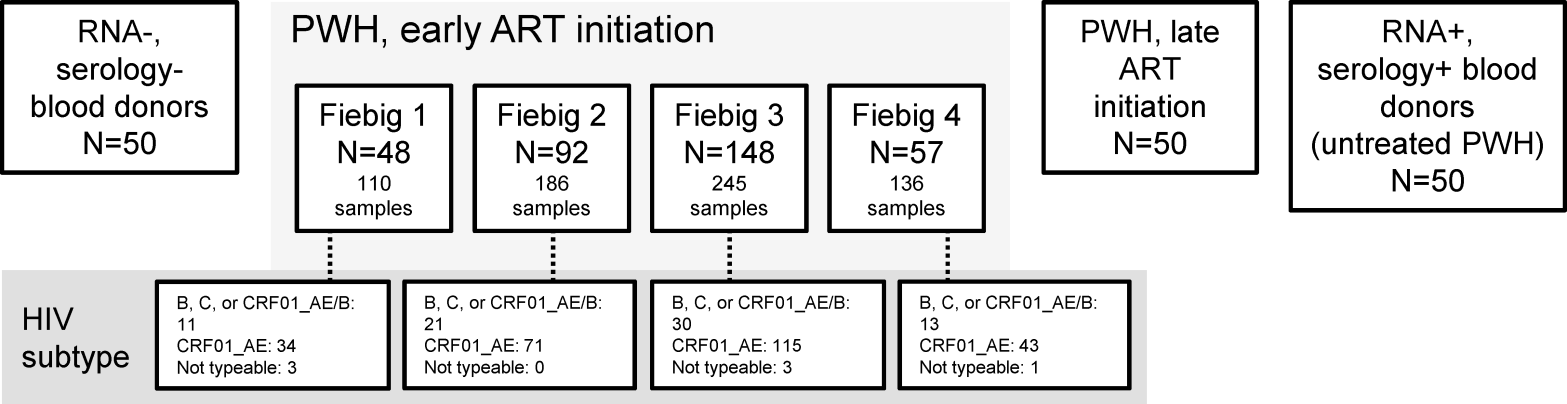
Panels and samples included in the study.

The RV254 early ART initiation group included 677 samples from 345 participants, of whom 48, 92, 148, and 57 started ART at Fiebig stages 1, 2, 3, and 4, respectively (Fig. 1). Only seven participants from this group had non-typeable HIV-1 subtypes, and most (76%) had the recombinant form CRF01_AE, consistent with HIV epidemiology in Thailand (23). The latest available samples from participants in this group were collected between 96 and 480 weeks after ART initiation (median 336 weeks). In the late-ART initiation group, samples were collected between 98 and 1,503 weeks after ART initiation (median 849 weeks).

### Ag/Ab test reactivity

The percentages of reactive Ag/Ab tests observed in the early-ART initiation group are described in Table 1 by Fiebig stage at ART initiation and by strata of the HIV-1 subtype. Among participants starting ART at Fiebig 1, the overall reactivity was 27% for Alinity and 60% for VITROS, with subtype-specific reactivity showing similar estimates and overlapping 95% confidence intervals (*CIs*). For participants starting ART at Fiebig 2, the reactivity was 73% for Alinity and 98% for VITROS. Among participants starting ART at Fiebig 3, the reactivity was 92% and 99% for Alinity and VITROS, respectively, whereas among those starting ART at Fiebig 4, the reactivity was 88% for Alinity and 97% for VITROS. Because the RV254 samples were collected in Thailand where the recombinant virus CRF01_AE is common, we hypothesized that lower sensitivity for Ab detection for those initiating ART early in infection might be related to the infecting virus subtype. There was no difference in detection of CRF01_AE relative to subtypes B, C, or CRF01_AE/B for those initiating ART in Fiebig stages 1 or 3. Alinity reactivity in those initiating ART in Fiebig stage 2 was somewhat lower for CRF01_AE (67%) relative to subtypes B, C, or CRF01_AE/B (91%), although 95% *CIs* overlapped. Overall, serological detection of HIV in long-term ART-suppressed PWH was less sensitive in those who initiated ART very early following infection, and there was no statistically significant effect of HIV subtype on assay sensitivity.

**TABLE 1:** Sensitivity of Alinity and VITROS assays for HIV serologic detection by subtype and Fiebig stage at ART initiation among RV254 participants

Participants	Fiebig 1	Fiebig 2	Fiebig 3	Fiebig 4
All subtype samples (*N* = 345 participants)	*N* = 48	*N* = 92	*N* = 148	*N* = 57
Alinity % (95% *CI*)	27% (15–42)	73% (63–82)	92% (86–96)	88% (76–95)
VITROS % (95% *CI*)	60% (45–74)	98% (92–100)	99% (95–100)	97% (88–100)
Subtypes B + C + CRF01_AE/B samples (*N* = 75 participants)	*N* = 11	*N* = 21	*N* = 30	*N* = 13
Alinity % (95% *CI*)	27% (6–61)	91% (70–99)	99% (88–100)	83% (55–98)
VITROS % (95% *CI*)	59% (31–89)	95% (76–100)	100% (88–100)	94% (64–100)
Subtype CRF01_AE % samples (*N* = 263 participants)	*N* = 34	*N* = 71	*N* = 115	*N* = 43
Alinity % (95% *CI*)	28% (15–47)	67% (55–78)	90% (84–95)	92 (81–99)
VITROS % (95% *CI*)	65% (46–80)	99% (92–100)	99 (95–100)	100% (92–100)

### S/CO values and correlations with time since ART initiation

**Fig 2:**
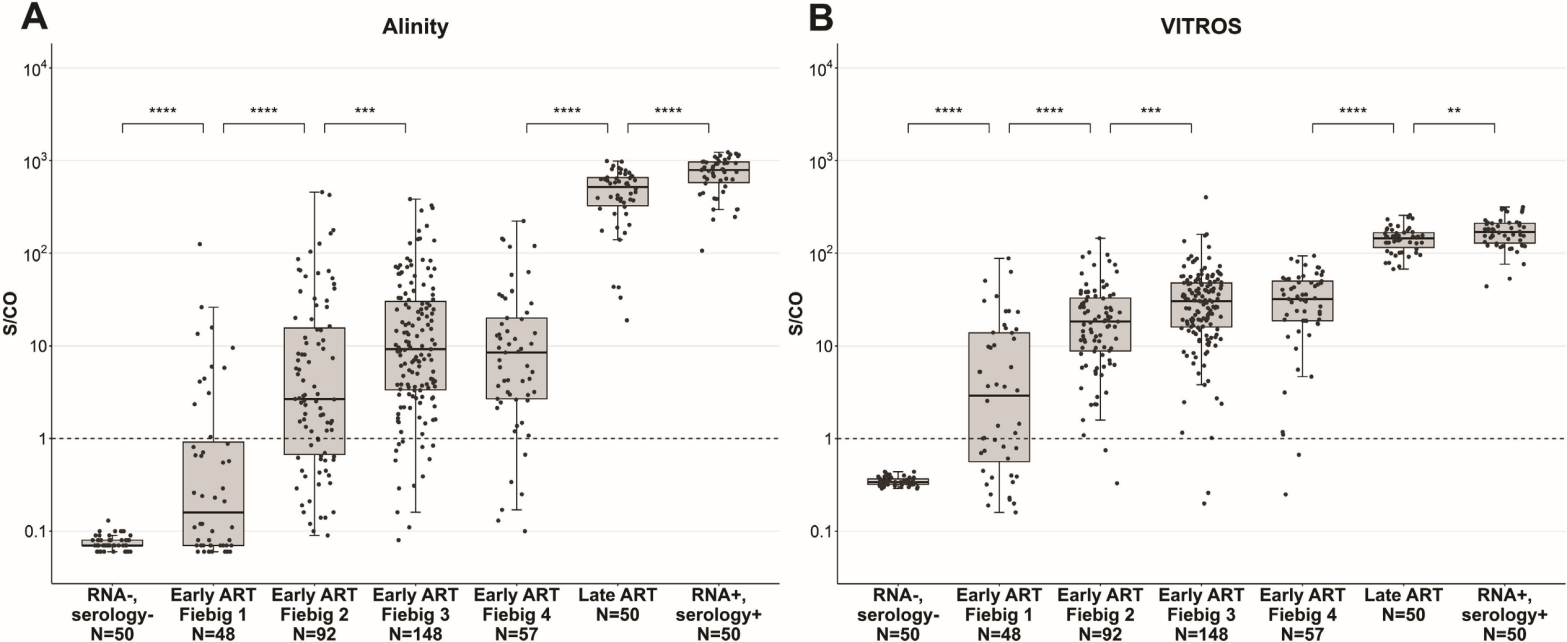
S/CO values on Ag/Ab combo assays by group. (**A**) Abbott Alinity assay. (**B**) Ortho VITROS assay. Samples from 345 RV254 participants are restricted to the latest available time points. **P < 0.01 , ***P < 0.001, and ****P < 0.0001.

As expected, all 50 samples from the RNA−/ serology− group were nonreactive in both Alinity and VITROS testing, and all samples from the late ART initiation and from the RNA+/serology+ groups were reactive in both Alinity and VITROS testing (Fig. 2). To determine if time from ART initiation influenced the Ab signal, we tested the last available sample from each of the 345 participants in the RV254 trial as well as the comparator groups. The RV254 samples tested were collected at a median of 336 weeks after ART initiation (IQR 336–336, range 96–480), and the time from ART initiation did not vary significantly between Fiebig stage groups. We found a progressive and statistically significant increase in median S/CO values comparing the study groups from participants starting ART at Fiebig stages 1 through 3 with no further increase with ART initiation at Fiebig stage 4 in the early-ART initiation group (Fig. 2). In terms of the distribution of nonreactive samples across all time points for RV254, Alinity had 28% nonreactive samples (48%, 28%, 11%, and 12% initiating ART in Fiebig stages 1, 2, 3, and 4, respectively) while VITROS had 8% nonreactive samples (78%, 5%, 5%, and 13% initiating ART in Fiebig stages 1, 2, 3, and 4, respectively).

Those initiating ART later in infection showed higher S/CO values than those initiating ART in Fiebig stage 3/4. The group with the highest reactivity was the RNA+ and serology+ blood donor group, who presumably had untreated HIV infection.

**Fig 3:**
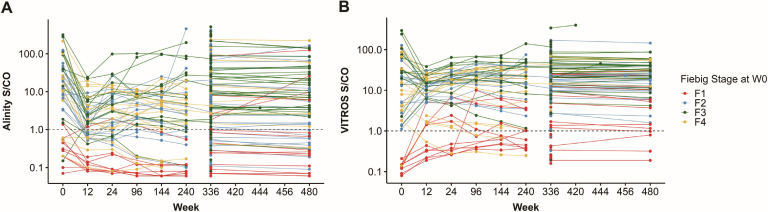
S/CO values on Ag/Ab combo assays over time among RV254 participants by Fiebig stage at ART initiation. (**A**) Abbott Alinity assay. (**B**) Ortho VITROS assay. Week 0 was defined as ART initiation. For time points 0–24 weeks, 55 participants were included, with 10 starting ART at Fiebig stage 1, 15 at stage 2, 15 at stage 3, and 15 at stage 4. Three hundred participants' samples were available at week 336, and 75 participants' samples were available at week 480. X-axis is not to scale.

**Fig 4:**
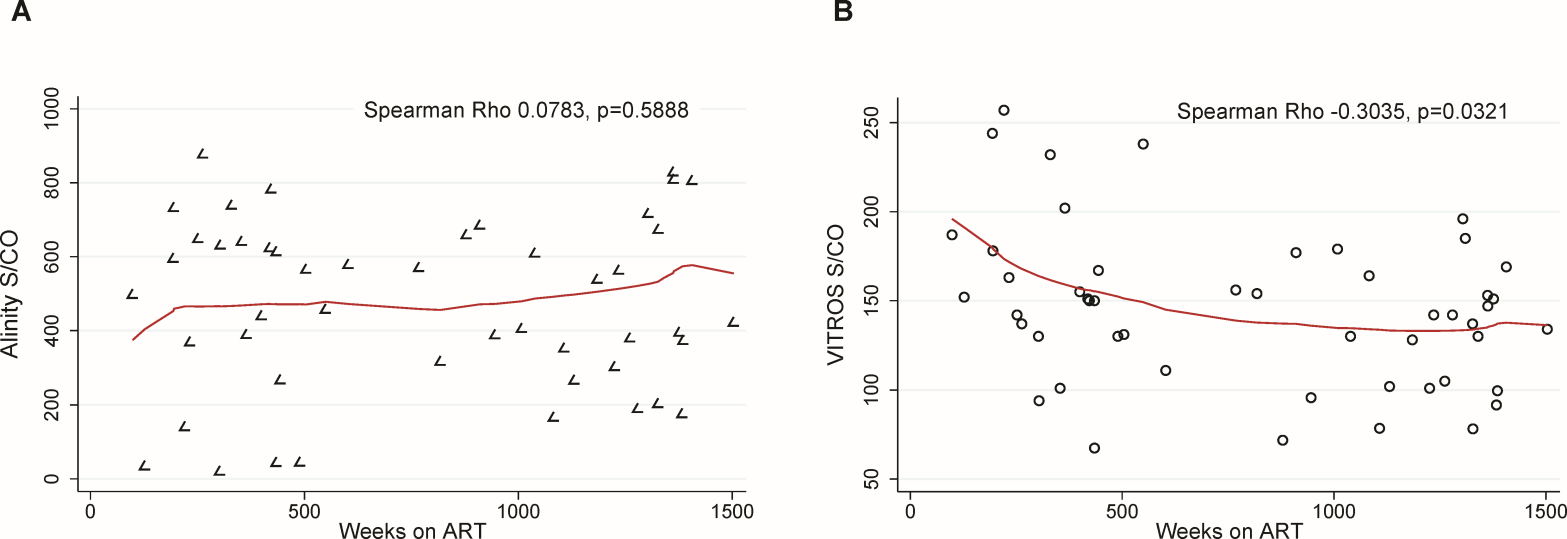
Correlations between time on antiretroviral treatment and S/CO values in the late- ART initiation group. (**A**) Abbott Alinity assay. (**B**) Ortho VITROS assay. Line represents best fit.

Figure 3 shows the individual evolution of S/CO values over time after ART initiation for participants in the early ART initiation group, as well as all available samples from the 336 and 480 week time points. Nonreactivity was more common but not exclusive in those starting ART at Fiebig 1 across all time points. There was not a significant change in assay signal over time across all 480 weeks of study. However, there was a significant drop in signal from week 0 to 12 for both assays (*P* < 0.01), and a 5%–10% decrease in values from week 336 to 480 that was not statistically significant. In the late ART initiation group, we found a statistically significant, inverse correlation between time since ART initiation and S/CO values for VITROS, with a more consistent negative relationship for time points <500 weeks (Fig. 4). Finally, we did not observe a significant correlation between time since ART initiation and S/CO values for Alinity.

### Sensitivity and specificity of Alinity at different test cutoffs

**Fig 5:**
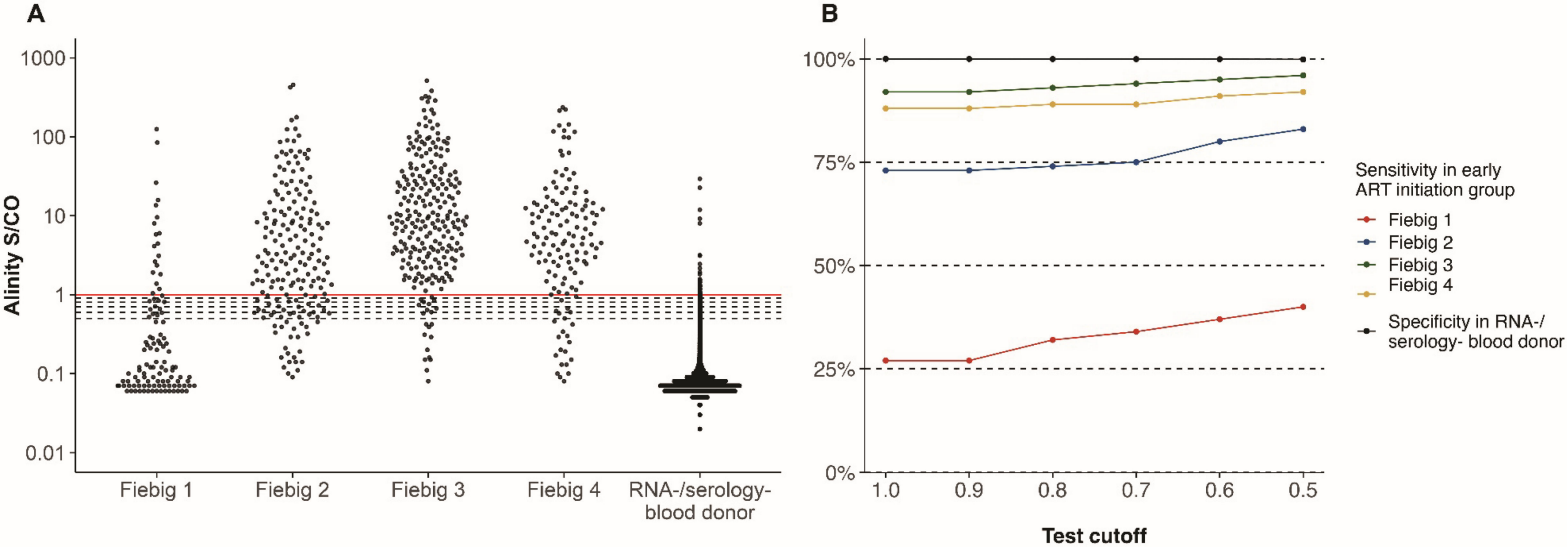
Alinity S/CO distributions, sensitivity, and specificity at different test cutoffs. (**A**) Alinity S/CO distributions in the early-ART initiation group by Fiebig stage at ART initiation, and in RNA−/serology− blood donations. The red line indicates the current reactivity cutoff; dashed lines indicate lower cutoffs at ≥0.9, 0.8, 0.7, 0.6, and 0.5 S/CO. (**B**) Alinity sensitivity in the early-ART initiation group by Fiebig stage at ART initiation and specificity in RNA−/serology− blood donations at different test cutoffs. S/CO: signal-to-cutoff; ART, antiretroviral treatment.

Given Alinity’s wide dynamic range and lower sensitivity in detecting HIV seropositivity among samples from the early-ART initiation group, we explored the impact of different reactivity cutoffs on the assay’s sensitivity, stratified by Fiebig stage at ART initiation. We also explored the impact of different reactivity cutoffs on specificity using as a reference the S/CO readouts obtained from routine donation screening of a large sample of RNA−/serology− blood donors. A reduction in the test cutoff from 1.0 to 0.5 increased the sensitivity from 27% to 40% for PWH, starting ART at Fiebig 1 and from 73% to 83% for PWH starting ART at Fiebig 2 (Table 2; Fig. 5). The impact on test sensitivity was more modest for those initiating ART at Fiebig stages 3 (92% to 96%) and 4 (88% to 92%). The specificity decreased from 99.99% to 99.87% with the same reduction in test cutoff.

**TABLE 2:** Alinity sensitivity and specificity at different test cutoffs

	Sensitivity among RV254 samples				Specificity in RNA−/serology− BD	
Test cutoff	Fiebig 1, *N* = 48	Fiebig 2, *N* = 92	Fiebig 3, *N* = 148	Fiebig 4, *N* = 57	*N* = 373,215 (95% *CI*)	# false-positive/100K
≥1.0	27%	73%	92%	88%	99.99% (99.98–99.99)	45
≥0.9	27%	73%	92%	88%	99.98% (99.97–99.98)	92
≥0.8	32%	74%	93%	89%	99.96% (99.95–99.97)	146
≥0.7	34%	75%	94%	89%	99.95% (99.94–99.95)	203
≥0.6	37%	80%	95%	91%	99.92% (99.91–99.92)	315
≥0.5	40%	83%	96%	92%	99.87% (99.86–99.88)	478

## DISCUSSION

We observed that earlier ART initiation was associated with progressively lower S/CO levels in commercial Ag/Ab assays, with a test sensitivity of 27% for Alinity and 60% for VITROS for samples obtained from long-term ART-suppressed PWH starting ART at Fiebig stage 1. Differences in serologic reactivity related to HIV subtype did not explain our findings, although Alinity positivity in samples from participants initiating ART at Fiebig stage 2 was somewhat lower for CRF01_AE (67%) than for other HIV subtypes (91%). We also observed significantly higher S/CO values in samples from RNA+/serology+ blood donors, who presumably had untreated infection. We observed no strong evidence of S/CO waning at later time points after ART initiation in the early- ART initiation group.

The highest S/CO levels observed in samples from blood donors with presumably untreated infection compared to participants on ART are aligned with the observation that effective suppression of HIV replication is associated with a decrease in HIV-specific Ab levels (12, 24). This finding was also supported by the inverse correlation between time on ART and S/CO titers observed for VITROS testing in samples from the late-ART initiation group and by the progressively lower S/CO levels associated with earlier ART initiation. Early ART initiation among PWH has been associated with a blunted Ab reactivity in studies including both pediatric and adult populations, presumably due to the limited antigenic stimulus resulting from the rapid suppression of HIV replication. However, prior studies were limited by a shorter follow-up time after ART initiation and by the adoption of less sensitive serologic assays (4, 8–10). Using Ag/Ab HIV combination assays that are currently approved for either blood donation screening or diagnostic testing, we observed that although early treatment was associated with lower S/CO titers compared to late treatment, S/CO levels remain relatively stable up to 480 weeks after early ART initiation, with variations restricted to the first weeks following early ART onset, including some participants presenting persistently nonreactive results. While it is reassuring that current Ag/Ab HIV combination assays are more resilient to waning patterns previously observed with less sensitive assays, our findings highlight that early ART may be associated with false-negative serologic test results even when state-of-the-art assays are used.

HIV subtypes are characterized by genetic variations that may influence HIV transmissibility (25), disease progression (26), and treatment response (27). In addition, different HIV subtypes may also affect Ab responses, potentially impacting the accuracy of serologic tests. For this reason, serologic tests are purposely evaluated regarding their ability to ascertain HIV status in sample panels including multiple HIV subtypes (13–15). CRF01_AE is a recombinant form of HIV-1, which originated in Central Africa, and is highly prevalent in Southeast and East Asia (28). CRF01_AE has been associated with higher viral load and faster disease progression (29–31), with possible differences in Ab evolution that are still incompletely understood (32). In our study, a signal for a potential subtype-related effect on serologic reactivity was observed only among participants starting ART during Fiebig 2 stage, a time point when the viral load is relatively high, but Ab responses are still poorly developed.

Differences in the performance of Alinity and VITROS in detecting HIV Ag/Ab reactivity in samples from the early- ART initiation group were noted. While both Alinity and VITROS detect p24 Ag and Abs to HIV types 1 and 2 envelope, the exact antigenic composition of each assay is not publicly disclosed; therefore, the specific mechanism underlying these performance differences cannot be determined. Nonetheless, ART is initiated after Ab responses are fully established for the vast majority of PWH, and both tests have very high sensitivity and specificity in this context.

Our study had limitations. Participants from the early-ART initiation group were recruited in Thailand, and subtype analysis confirmed the higher prevalence of CRF01-AE, as previously described (33); in contrast, participants from the late-ART initiation group and RNA+/serology+ blood donors were all from Brazil, where subtype B is the most prevalent (34). However, the data presented in Table 1 do not show a strong effect of virus subtype on assay performance, so the finding of lower assay sensitivity in those who initiate ART very early in treatment appears valid. Subtype testing for 78% of the samples in the early-ART initiation group was performed with MSSP rather than by full-length sequencing. Ag/Ab tests were used outside of package insert requirements to test clinical samples collected for diagnostic purposes using the Alinity s HIV Ag/Ab Combo Reagent Kit, which is only approved for blood donation screening under specific sample handling requirements including storage at a combination of 15°C to 30°C and 2°C to 8°C for <14 days inclusive of shipping time and storage at ≤ −20°C for <3 months with <6 freeze/thaw cycles. It is difficult to guarantee that study samples collected, processed, stored in different countries and shipped across different sites/countries under various procedures will meet package insert requirements. On the other end, the package insert for the VITROS Immunodiagnostic Products HIV Combo Reagent Pack assay, which is only approved for Diagnostic, includes specimen handling requirements that are more stringent than the Alinity assay. Indeed, samples may be stored for up to 24 h at room temperature or 7 days at 2°C–8°C, and if not tested within this time frame, they should be stored at −20 °C and may be subjected to up to five freeze-thaw cycles. Beyond variations in sample collection, processing, storage, and shipping procedures, testing was performed at different laboratories, with one additional freeze-thaw cycle prior to Alinity testing relative to VITROS for most samples. Samples from the early-ART initiation group had been stored at ≤−20°C for a varying period, many of which were beyond Alinity assay’s labeled claim. Despite these limitations, our study included a large sample of long-term treated PWH, including participants with well-documented early ART initiation, and prior study has demonstrated stability of anti-HIV Abs stored at −20°C for up to 18 years (35).

Our findings suggest potential limitations for HIV infection ascertainment among long-term ART-suppressed PWH who started ART at early infection stages using clinical samples collected for diagnostic purposes with the currently FDA-approved blood donation screening serologic test Alinity s HIV Ag/Ab Combo Reagent kit assay, particularly for those starting ART at Fiebig stages 1 or 2. Reassuringly, it is rare that a PWH starts ART at such early stages and even more improbable that this situation occurs for a blood donor who fails to disclose their HIV status and ART intake during the donor history questionnaire. Indeed, blood donors are specifically asked about ever testing positive for HIV infection, taking any medications to treat HIV infection, taking any medication in the deferral list of drugs that includes specific HIV prevention and treatment drugs, and having taken any medication to prevent oral transmission of HIV over the past 3 months or through injection over the past 2 years. However, given that some donors do not report accurately their HIV status and HIV drug intake (3, 36), further research to identify limitations of testing and opportunities to improve sensitivity remains important.

## Data Availability

Currently, the datasets used and/or analyzed during the study are available from the authors on reasonable request.
